# Supplementary material for: Proteomics of Multiple Sclerosis: Inherent Issues in Defining the Pathoetiology and Identifying (Early) Biomarkers
Source: Int J Mol Sci. 2021 Jul 9;22(14):7377. doi: 10.3390/ijms22147377 (PMC8306353; doi:10.3390/ijms22147377)
Supplement: Supplementary file 1 [file ijms-22-07377-s001.zip › Supplemetary/Sen et al_Supplementary Figure and Table.pdf]

Proteomics of Multiple Sclerosis: Inherent Issues in Defining the Pathoetiology and Identifying (Early) Biomarkers

Monokesh K. Sen [1,†], Mohammed S.M. Almuslehi [1,2], Peter J. Shortland [3], David A. Mahns [1] and Jens R. Coorssen [4,*]

[1]School of Medicine, Western Sydney University, Locked Bag 1797, Penrith, NSW 2751, Australia; monokesh.sen@westernsydney.edu.au (M.K.S.); m.almuslehi@westernsydney.edu.au (M.S.M.A.); d.mahns@westernsydney.edu.au (D.A.M.)
[2]Department of Physiology & Pharmacology, College of Veterinary Medicine, University of Diyala, Baqubah 32001, Diyala, Iraq
[3]School of Science, Western Sydney University, Locked Bag 1797, Penrith, NSW 2751, Australia; p.shortland@westernsydney.edu.au
[4]Departments of Health Sciences and Biological Sciences, Faculties of Applied Health Sciences and Mathematics & Science, Brock University, St. Catharines, ON L2S 3A1, Canada
*Correspondence: jcoorssen@brocku.ca
†Current address: Charles Perkins Centre, School of Medical Sciences, University of Sydney, Sydney, NSW 2006, Australia.

Supplementary Table S1: Examples of canonical proteins identified by bottom-up and proteoforms in top-down studies

Canonical proteins	Gene ID	Molecular function	Experimental group and sample analysed						Theoretical vs experimental MW and pI
			MS		EAE		CPZ		
			TD	BU	TD	BU	TD	BU	
Septin	Sept	Structural	↓[1]; blood	-	↑↓[2-5]; cerebrum, brain stem, spinal cord	↑↓[6,7]; spinal cord	↓[8]; cerebrum	-	41.5/6.1 vs 81/5.7
Tubulin	Tub	Structural	↑[1]; blood	-	↑↓[4,5,9]; cerebrum, brain stem, spinal cord	↑↓[6,7]; spinal cord	↓[10]; spleen	-	50.1/4.8 vs 28.2/5.3
Compleme nt (e.g. C3, C4)	C3	Immune response	↑↓[11-16]; CSF, blood, tear	↑↓[17-19]; CSF, blood, cerebrum, cerebellum, brain stem, spinal cord	-	↑↓[6,20-22]; spinal cord	↓[10]; blood	-	31.3/4.8 vs 34.6/4.4
Glial fibrillary acidic protein	Gfap	Immune response	-	↑[23]; cerebrum	↑[2,5,9,24]; brain stem, spinal cord	↑[6,7]; spinal cord	↑[8]; cerebrum	↑[25]; cerebrum	49.8/5.2 vs 83.7/5.0
Protein disulfide-isomerase	Pdia	Molecular chaperone	↑[1]; blood	↓[26]; blood	↑↓[5,9,24]; brain stem, spinal cord	↑[6]; spinal cord	↓[10]; spleen	-	58.6/4.8 vs 63.7/4.7
Calreticulin	Calr	Molecular chaperone	-	-	↓[2,3]; cerebrum, spinal cord	↑[6,7]; spinal cord	↑[8]; cerebrum	-	47.9/4.3 vs 155/4.4
Hexokinase	Hk	Metabolic	-	-	-	↑↓[6,7]; spinal cord	↓[8]; cerebrum	-	101.8/6.2 vs 220/6.4
Aconitate hydratase	Aco2	Metabolic	-	-	↑[5]; brain stem	↓[7]; spinal cord	↑[8]; cerebrum	-	85.4/8.1 vs 182.9/8.2
Dynamin 1	Dnm 1	Endocytosis	-	-	↓[5]; spinal cord	↓[6,7]; spinal cord	↓[10]; cerebrum	-	98.1/7.6 vs 122.6/5.6
Dynamin 1	Dnm 1	Endocytosis	-	-	↓[5]; spinal cord	↓[6,7]; spinal cord	↑[8]; cerebrum	-	93.9/6.2 vs 200/6.2

Key: Theoretical vs experimental MW and pI are from two studies [8,10] as only these reported the critical data required to clearly identify proteoforms. Abbreviations: ↑, increase; ↓, decrease; MS, Multiple Sclerosis; EAE, Experimental Autoimmune Encephalomyelitis; CPZ, Cuprizone; TD, Top-down; BU, Bottom-up and -, not found/no research. Studies that mentioned only the presence of a protein without describing the magnitude of change (e.g. fold increase or decrease) compared to Controls are indicated with a ↑ sign. On the other hand, if a protein was described as absent, a ↓ sign is used, to maintain the consistency with other studies.

Dynamin 1 was identified with two different theoretical MW and pI (98.1/7.6 vs 93.9/6.2) between studies [8,10]. Partridge et al. [10] used SwissProt and LudwigNR databases (UniProt ID: P39053) to confirm the canonical protein identity whereas Sen et al. [8] used the MSPnr100 database (UniProt ID: A0A0J9YUE9) to identify Dynamin 1. Nonetheless, Partridge et al. [10] used pooled cortex (cerebrum) samples from 8-week-old female C57Bl/6 mice following 0.2% CPZ-feeding for 5 weeks, whereas Sen et al. [8] analysed individual cerebrum samples from 8-week-old male C57Bl/6 mice following 0.1 and 0.2% CPZ-feeding for 5 and 12 weeks. The data thus suggest that sex of the study animals, CPZ dose (or use of different databases) may have influenced which proteoforms were identified. However, other 2DE methodical aspects such as protein extraction, staining (both used cCBB) or LC-TMS analysis were similar between studies.

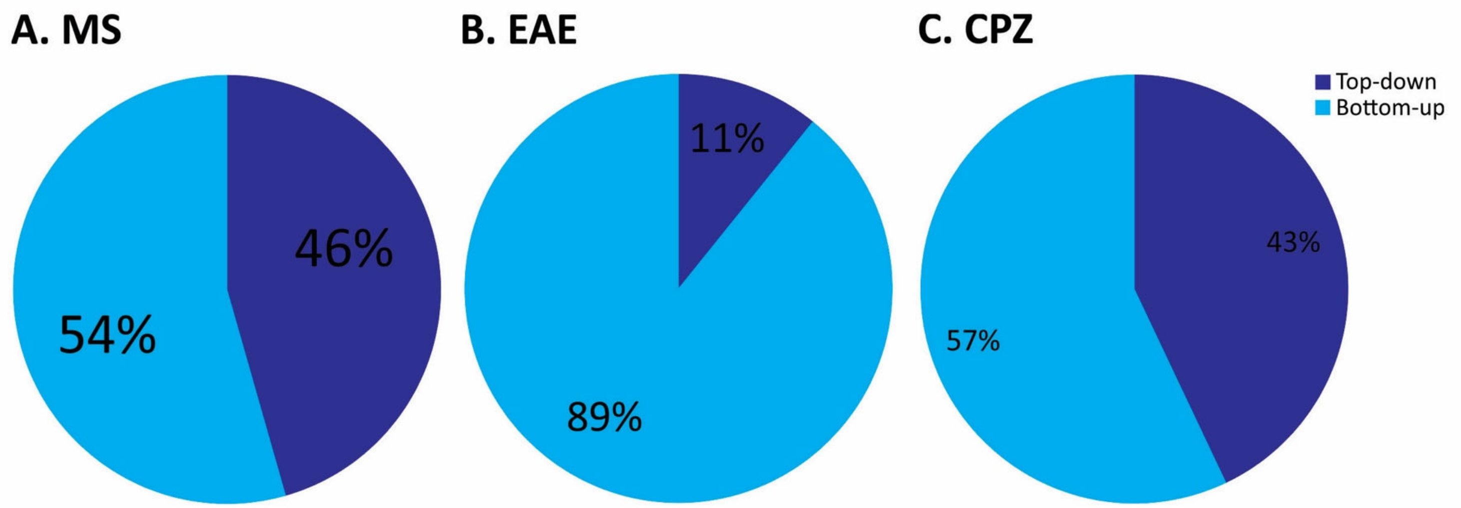

Supplementary Figure S1a: Percentage of canonical proteins identified using top-down and bottom-up approaches across MS (**A**), EAE (**B**), and CPZ (**C**). A full list of protein information, including proteomic approach, and instrument used to analyse samples, has been provided in **Table 3** and the **Supplementary Excel File S1**.

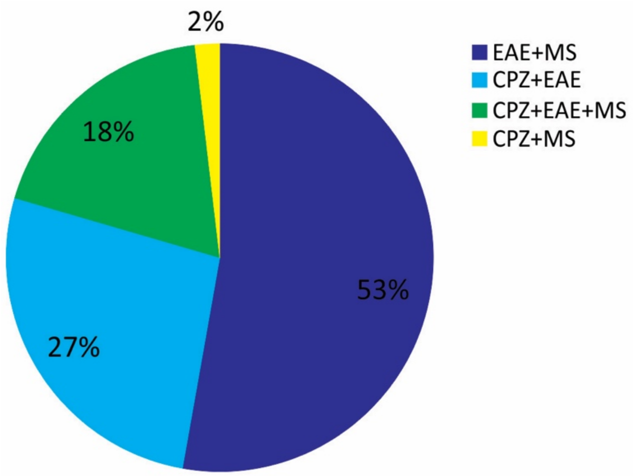

Supplementary Figure S1b: Similarities of canonical proteins (i.e. commonly identified proteins, **Table 3**) among different biological systems (MS, EAE, and CPZ).

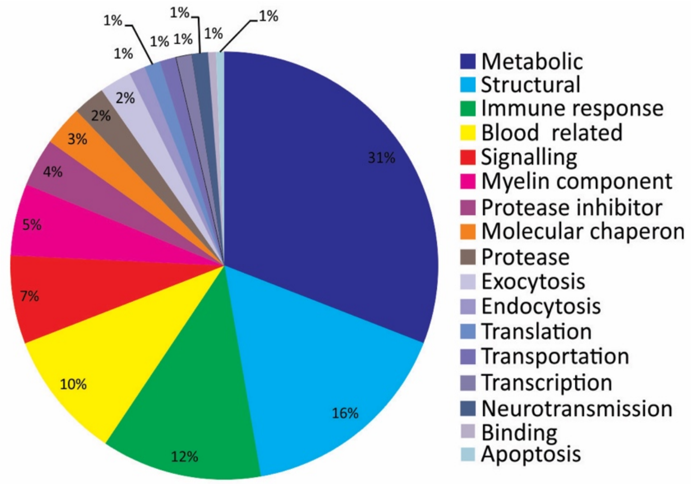

Supplementary Figure S1c: Molecular functions of commonly identified proteins (i.e. those identified canonical proteins changing in at least two or more biological systems (MS, EAE and CPZ); a full list of information is provided in **Table 3**.

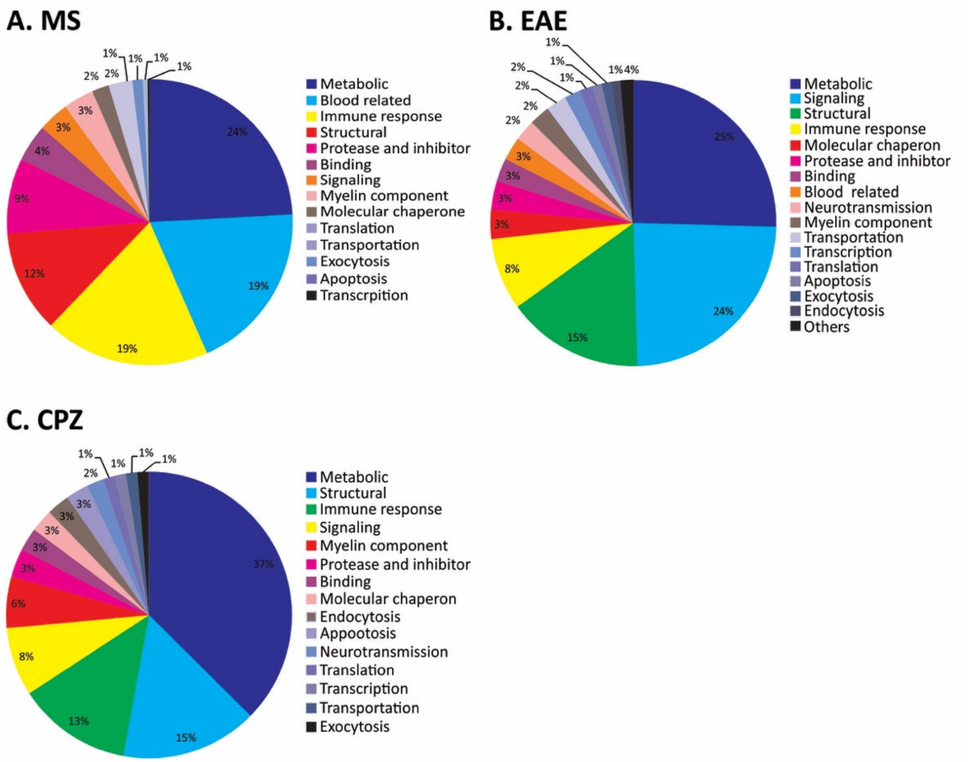

Supplementary Figure S1d: Comparison of the molecular functions of proteins identified in the three biological systems: **A**) MS, **B**) EAE and **C**) CPZ). A full list of canonical proteins and their individual molecular functions is provided in the **Supplementary Excel File S1**.

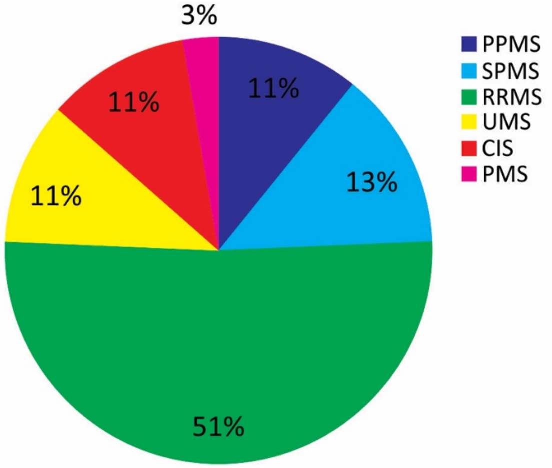

Supplementary Figure S1e: Canonical protein identifications among different phenotypes of MS. Detailed descriptions of canonical proteins that were found in different phenotypes (e.g. RRMS, PPMS) of MS are provided in **Table 4** and the **Supplementary Excel File S1**.

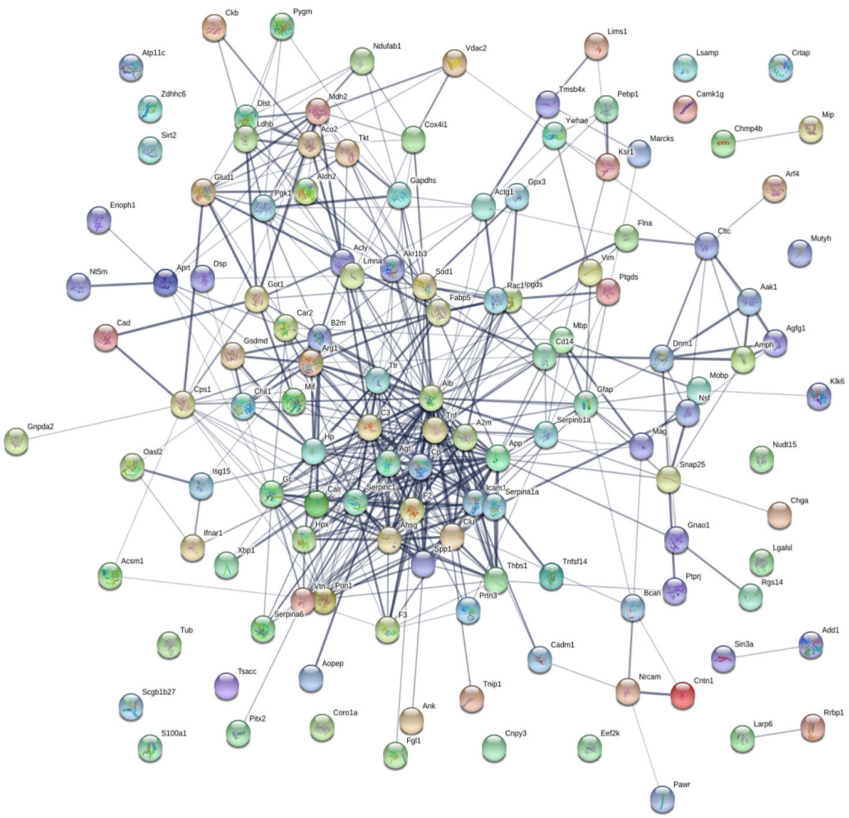

Supplementary Figure S1f: Protein-protein interactions of commonly identified canonical proteins (**Table 3**) as defined using STRING analysis. Here each node indicates the name of a gene ID (every protein has a gene ID) and the thickness of lines indicates the strength of interaction. This figure indicates complex interactions among proteins. A full list of proteins and their gene IDs is provided in the **Supplementary Excel File S1**.

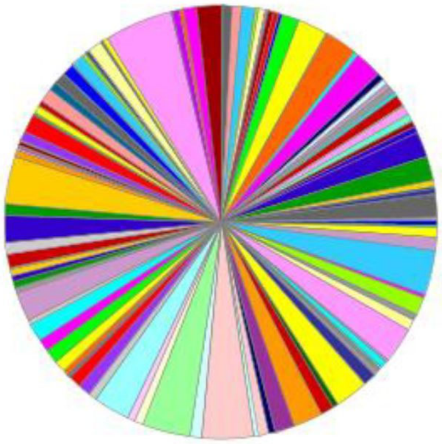

- 2-arachidonoylglycerol biosynthesis (P05726)
- 5-Hydroxytryptamine biosynthesis (P04371)
- 5-Hydroxytryptamine degredation (P04372)
- 5HT1 type receptor mediated signaling pathway (P04373)
- 5HT2 type receptor mediated signaling pathway (P04374)
- 5HT3 type receptor mediated signaling pathway (P04375)
- 5HT4 type receptor mediated signaling pathway (P04376)
- ALP23B signaling pathway (P06209)
- ATP synthesis (P02721)
- Activin beta signaling pathway (P06210)
- Adenine and hypoxanthine salvage pathway (P02723)
- Adrenaline and noradrenaline biosynthesis (P00001)
- Allantoin degradation (P02725)
- Alpha adrenergic receptor signaling pathway (P00002)
- Alzheimer disease-amyloid secretase pathway (P00003)
- Alzheimer disease-presenilin pathway (P00004)
- Aminobutyrate degradation (P02726)
- Angiogenesis (P00005)
- Angiotensin II-stimulated signaling through G proteins and beta-arrestin (P05911)
- Apoptosis signaling pathway (P00006)
- Arginine biosynthesis (P02728)
- Ascorbate degradation (P02729)
- Asparagine and aspartate biosynthesis (P02730)
- Axon guidance mediated by Slit/Robo (P00008)
- Axon guidance mediated by netrin (P00009)

- Axon guidance mediated by semaphorins (P00007)
- B cell activation (P00010)
- BMP/activin signaling pathway-drosophila (P06211)
- Beta1 adrenergic receptor signaling pathway (P04377)
- Beta2 adrenergic receptor signaling pathway (P04378)
- Beta3 adrenergic receptor signaling pathway (P04379)
- Blood coagulation (P00011)
- CCKR signaling map (P06959)
- Cadherin signaling pathway (P00012)
- Cell cycle (P00013)
- Circadian clock system (P00015)
- Cortocotropin releasing factor receptor signaling pathway (P04380)
- Cytoskeletal regulation by Rho GTPase (P00016)
- DPP signaling pathway (P06213)
- DPP-SCW signaling pathway (P06212)
- De novo purine biosynthesis (P02738)
- De novo pyrimidine deoxyribonucleotide biosynthesis (P02739)
- De novo pyrimidine ribonucleotides biosythesis (P02740)
- Dopamine receptor mediated signaling pathway (P05912)
- EGF receptor signaling pathway (P00018)
- Endogenous cannabinoid signaling (P05730)
- Endothelin signaling pathway (P00019)
- Enkephalin release (P05913)
- FAS signaling pathway (P00020)
- FGF signaling pathway (P00021)
- Fructose galactose metabolism (P02744)
- GABA-B receptor II signaling (P05731)
- GBB signaling pathway (P06214)
- Gamma-aminobutyric acid synthesis (P04384)
- General transcription by RNA polymerase I (P00022)
- General transcription regulation (P00023)
- Glutamine glutamate conversion (P02745)
- Glycolysis (P00024)
- Gonadotropin-releasing hormone receptor pathway (P06664)
- Hedgehog signaling pathway (P00025)
- Heme biosynthesis (P02746)
- Heterotrimeric G-protein signaling pathway-Gi alpha and Gs alpha mediated pathway (P00026)
- Heterotrimeric G-protein signaling pathway-Gq alpha and Go alpha mediated pathway (P00027)
- Heterotrimeric G-protein signaling pathway-rod outer segment phototransduction (P00028)

- Histamine H1 receptor mediated signaling pathway (P04385)
- Histamine H2 receptor mediated signaling pathway (P04386)
- Histamine synthesis (P04387)
- Huntington disease (P00029)
- Hypoxia response via HIF activation (P00030)
- Inflammation mediated by chemokine and cytokine signaling pathway (P00031)
- Insulin/IGF pathway-mitogen activated protein kinase kinase/MAP kinase cascade (P00032)
- Insulin/IGF pathway-protein kinase B signaling cascade (P00033)
- Integrin signalling pathway (P00034)
- Interferon-gamma signaling pathway (P00035)
- Interleukin signaling pathway (P00036)
- Ionotropic glutamate receptor pathway (P00037)
- JAK/STAT signaling pathway (P00038)
- Lysine biosynthesis (P02751)
- MYO signaling pathway (P06215)
- Metabotropic glutamate receptor group I pathway (P00041)
- Metabotropic glutamate receptor group II pathway (P00040)
- Metabotropic glutamate receptor group III pathway (P00039)
- Muscarinic acetylcholine receptor 1 and 3 signaling pathway (P00042)
- Muscarinic acetylcholine receptor 2 and 4 signaling pathway (P00043)
- Nicotine pharmacodynamics pathway (P06587)
- Nicotinic acetylcholine receptor signaling pathway (P00044)
- Notch signaling pathway (P00045)
- Opioid prodynorphin pathway (P05916)
- Opioid proenkephalin pathway (P05915)
- Opioid proopiomelanocortin pathway (P05917)
- Ornithine degradation (P02758)
- Oxidative stress response (P00046)
- Oxytocin receptor mediated signaling pathway (P04391)
- P53 pathway feedback loops 1 (P04392)
- PDGF signaling pathway (P00047)
- PI3 kinase pathway (P00048)
- Parkinson disease (P00049)
- Pentose phosphate pathway (P02762)
- Phenylethylamine degradation (P02766)
- Plasminogen activating cascade (P00050)
- Purine metabolism (P02769)
- Pyridoxal phosphate salvage pathway (P02770)
- Pyridoxal-5-phosphate biosynthesis (P02759)
- Pyrimidine Metabolism (P02771)

- Pyruvate metabolism (P02772)
- Ras Pathway (P04393)
- SCW signaling pathway (P06216)
- Salvage pyrimidine ribonucleotides (P02775)
- Serine glycine biosynthesis (P02776)
- Synaptic vesicle trafficking (P05734)
- T cell activation (P00053)
- TCA cycle (P00051)
- TGF-beta signaling pathway (P00052)
- Thiamin biosynthesis (P02779)
- Thyrotropin-releasing hormone receptor signaling pathway (P04394)
- Toll pathway-drosophila (P06217)
- Toll receptor signaling pathway (P00054)
- Transcription regulation by bZIP transcription factor (P00055)
- Tyrosine biosynthesis (P02784)
- Ubiquitin proteasome pathway (P00060)
- VEGF signaling pathway (P00056)
- Vasopressin synthesis (P04395)
- Vitamin B6 biosynthesis (P02786)
- Vitamin B6 metabolism (P02787)
- Vitamin D metabolism and pathway (P04396)
- Wnt signaling pathway (P00057)
- Xanthine and guanine salvage pathway (P02788)
- p38 MAPK pathway (P05918)
- p53 pathway by glucose deprivation (P04397)
- p53 pathway feedback loops 2 (P04398)
- p53 pathway (P00059)

Supplementary Figure S1g: PANTHER (Protein analysis through evolutionary relationships) analysis of commonly identified canonical proteins. Gene ID was used to investigate molecular pathways in PANTHER. A full list of proteins and their gene IDs is provided in the **Supplementary Excel File S1**.

References

1. De Masi, R.; Vergara, D.; Pasca, S., et al. PBMCs protein expression profile in relapsing IFN-treated multiple sclerosis: A pilot study on relation to clinical findings and brain atrophy. *J Neuroimmunol* **2009**, *210*, 80-86, doi:10.1016/j.jneuroim.2009.03.002.
2. Fazeli, A.S.; Nasrabadi, D.; Pouya, A., et al. Proteome analysis of post-transplantation recovery mechanisms of an EAE model of multiple sclerosis treated with embryonic stem cell-derived neural precursors. *J Proteomics* **2013**, *94*, 437-450, doi:10.1016/j.jprot.2013.06.008.
3. Fazeli, A.S.; Nasrabadi, D.; Sanati, M.H., et al. Proteome analysis of brain in murine experimental autoimmune encephalomyelitis. *Proteomics* **2010**, *10*, 2822-2832, doi:10.1002/pmic.200900507.
4. Jastorff, A.M.; Haegler, K.; Maccarrone, G., et al. Regulation of proteins mediating neurodegeneration in experimental autoimmune encephalomyelitis and multiple sclerosis. *Proteomics Clin Appl* **2009**, *3*, 1273-1287, doi:10.1002/prca.200800155.
5. Vanheel, A.; Daniels, R.; Plaisance, S., et al. Identification of protein networks involved in the disease course of experimental autoimmune encephalomyelitis, an animal model of multiple sclerosis. *PLoS One* **2012**, *7*, e35544, doi:10.1371/journal.pone.0035544.
6. Hasan, M.; Min, H.; Rahaman, K.A., et al. Quantitative Proteome Analysis of Brain Subregions and Spinal Cord from Experimental Autoimmune Encephalomyelitis Mice by TMT-Based Mass Spectrometry. *Proteomics* **2019**, *19*, e1800355, doi:10.1002/pmic.201800355.
7. Jain, M.R.; Li, Q.; Liu, T., et al. Proteomic Identification of Immunoproteasome Accumulation in Formalin-Fixed Rodent Spinal Cords with Experimental Autoimmune Encephalomyelitis. *J Proteome Res* **2012**, *11*, 1791-1803, doi:10.1021/pr201043u.
8. Sen, M.K.; Almuslehi, M.S.M.; Gyengesi, E., et al. Suppression of the Peripheral Immune System Limits the Central Immune Response Following Cuprizone-Feeding: Relevance to Modelling Multiple Sclerosis. *Cells* **2019**, *8*, 1314.
9. Farias, A.S.; Martins-de-Souza, D.; Guimaraes, L., et al. Proteome analysis of spinal cord during the clinical course of monophasic experimental autoimmune encephalomyelitis. *Proteomics* **2012**, *12*, 2656-2662, doi:10.1002/pmic.201200044.
10. Partridge, M.A.; Gopinath, S.; Myers, S.J., et al. An initial top-down proteomic analysis of the standard cuprizone mouse model of multiple sclerosis. *J Chem Biol* **2016**, *9*, 9-18, doi:10.1007/s12154-015-0138-0.
11. Dumont, D.; Noben, J.P.; Raus, J., et al. Proteomic analysis of cerebrospinal fluid from multiple sclerosis patients. *Proteomics* **2004**, *4*, 2117-2124, doi:10.1002/pmic.200300715.

12. Hammack, B.N.; Fung, K.Y.; Hunsucker, S.W., et al. Proteomic analysis of multiple sclerosis cerebrospinal fluid. *Mult Scler* **2004**, *10*, 245-260, doi:10.1191/1352458504ms1023oa.
13. Li, Y.; Qin, Z.; Yang, M., et al. Differential expression of complement proteins in cerebrospinal fluid from active multiple sclerosis patients. *J Cell Biochem* **2011**, *112*, 1930-1937, doi:10.1002/jcb.23113.
14. Liu, S.; Bai, S.; Qin, Z., et al. Quantitative proteomic analysis of the cerebrospinal fluid of patients with multiple sclerosis. *J Cell Mol Med* **2009**, *13*, 1586-1603, doi:10.1111/j.1582-4934.2009.00850.x.
15. Rithidech, K.N.; Honikel, L.; Milazzo, M., et al. Protein expression profiles in pediatric multiple sclerosis: potential biomarkers. *Mult Scler* **2009**, *15*, 455-464, doi:10.1177/1352458508100047.
16. Salvisberg, C.; Tajouri, N.; Hainard, A., et al. Exploring the human tear fluid: discovery of new biomarkers in multiple sclerosis. *Proteomics Clin Appl* **2014**, *8*, 185-194, doi:10.1002/prca.201300053.
17. Kroksveen, A.C.; Aasebo, E.; Vethe, H., et al. Discovery and initial verification of differentially abundant proteins between multiple sclerosis patients and controls using iTRAQ and SID-SRM. *J Proteomics* **2013**, *78*, 312-325, doi:10.1016/j.jprot.2012.09.037.
18. Kroksveen, A.C.; Guldbrandsen, A.; Vedeler, C., et al. Cerebrospinal fluid proteome comparison between multiple sclerosis patients and controls. *Acta Neurol Scand Suppl* **2012**, 10.1111/ane.12029, 90-96, doi:10.1111/ane.12029.
19. Teunissen, C.E.; Koel-Simmelink, M.J.; Pham, T.V., et al. Identification of biomarkers for diagnosis and progression of MS by MALDI-TOF mass spectrometry. *Mult Scler* **2011**, *17*, 838-850, doi:10.1177/1352458511399614.
20. Liu, T.; Donahue, K.C.; Hu, J., et al. Identification of differentially expressed proteins in experimental autoimmune encephalomyelitis (EAE) by proteomic analysis of the spinal cord. *J Proteome Res* **2007**, *6*, 2565-2575, doi:10.1021/pr070012k.
21. Dagley, L.F.; Croft, N.P.; Isserlin, R., et al. Discovery of novel disease-specific and membrane-associated candidate markers in a mouse model of multiple sclerosis. *Mol Cell Proteomics* **2014**, *13*, 679-700, doi:10.1074/mcp.M113.033340.
22. Stoop, M.P.; Rosenling, T.; Attali, A., et al. Minocycline effects on the cerebrospinal fluid proteome of experimental autoimmune encephalomyelitis rats. *J Proteome Res* **2012**, *11*, 4315-4325, doi:10.1021/pr300428e.
23. Ly, L.; Barnett, M.H.; Zheng, Y.Z., et al. Comprehensive tissue processing strategy for quantitative proteomics of formalin-fixed multiple sclerosis lesions. *J Proteome Res* **2011**, *10*, 4855-4868, doi:10.1021/pr200672n.
24. Linker, R.A.; Brechlin, P.; Jesse, S., et al. Proteome profiling in murine models of multiple sclerosis: identification of stage specific markers and culprits for tissue damage. *PLoS One* **2009**, *4*, e7624, doi:10.1371/journal.pone.0007624.
25. Werner, S.R.; Saha, J.K.; Broderick, C.L., et al. Proteomic analysis of demyelinated and remyelinating brain tissue following dietary cuprizone administration. *J Mol Neurosci* **2010**, *42*, 210-225, doi:10.1007/s12031-010-9354-9.
26. Berge, T.; Eriksson, A.; Brorson, I.S., et al. Quantitative proteomic analyses of CD4(+) and CD8(+) T cells reveal differentially expressed proteins in multiple sclerosis patients and healthy controls. *Clin Proteomics* **2019**, *16*, 19, doi:10.1186/s12014-019-9241-5.
